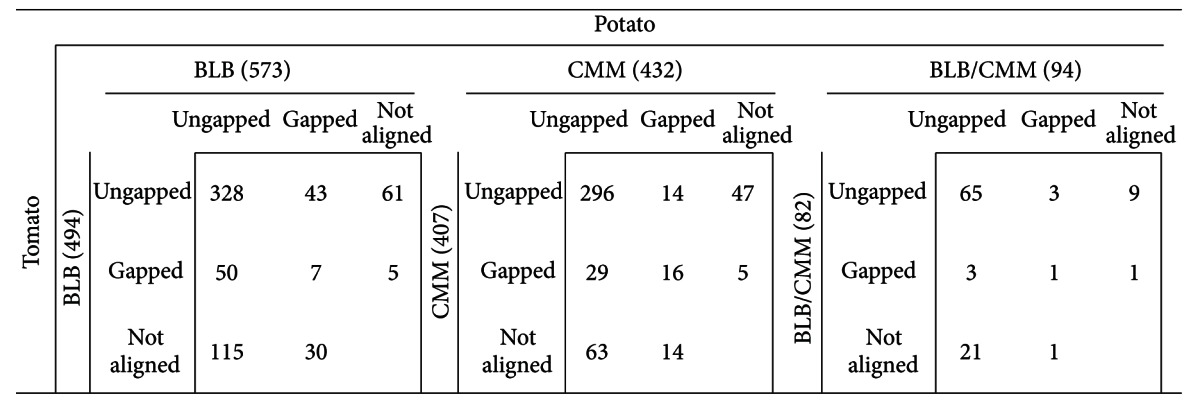# Genome Microscale Heterogeneity among Wild Potatoes Revealed by Diversity Arrays Technology Marker Sequences

**DOI:** 10.1155/2013/257218

**Published:** 2013-05-08

**Authors:** Alessandra Traini, Massimo Iorizzo, Harpartap Mann, James M. Bradeen, Domenico Carputo, Luigi Frusciante, Maria Luisa Chiusano

**Affiliations:** ^1^Department of Agricultural Sciences, University of Naples Federico II, Via Università 100, 80055 Portici, Naples, Italy; ^2^Department of Horticulture, University of Wisconsin-Madison, 1575 Linden Drive, Madison, WI 53706, USA; ^3^Department of Plant Pathology, University of Minnesota, 495 Borlaug Hall/1991 Upper Buford Circle, St. Paul, MN 55108, USA

## Abstract

Tuber-bearing potato species possess several genes that can be exploited to improve the genetic background of the cultivated potato *Solanum tuberosum*. Among them, *S. bulbocastanum* and *S. commersonii* are well known for their strong resistance to environmental stresses. However, scant information is available for these species in terms of genome organization, gene function, and regulatory networks. Consequently, genomic tools to assist breeding are meager, and efficient exploitation of these species has been limited so far. In this paper, we employed the reference genome sequences from cultivated potato and tomato and a collection of sequences of 1,423 potato Diversity Arrays Technology (DArT) markers that show polymorphic representation across the genomes of *S. bulbocastanum* and/or *S. commersonii* genotypes. Our results highlighted microscale genome sequence heterogeneity that may play a significant role in functional and structural divergence between related species. Our analytical approach provides knowledge of genome structural and sequence variability that could not be detected by transcriptome and proteome approaches.

## 1. Background

The subgenus *Potatoe* of the Solanaceae family includes approximately 188 tuber-bearing species [1]. They display large ecological adaptation encompassing several traits that are lacking in the commercial potato and useful for breeding [2]. Among wild potato species, *Solanum bulbocastanum* Dun. and *S. commersonii* Dun. ex Poir. have attracted the attention of researchers and breeders. *S. bulbocastanum* is a known source of resistance to late blight disease of potato, and four late blight resistance genes have been cloned from this species to date [3–7]. *S. commersonii* ranks first among *Solanums* in terms of cold tolerance and capacity to cold acclimate, and it is also a source of resistance to pathogens such as *Ralstonia solanacearum* and *Pectobacterium carotovorum* [8, 9]. *S. bulbocastanum* and *S. commersonii* are among approximately 20 diploid potato species classified as superseries *Stellata* by Hawkes [10]. Despite their importance as sources of genes for crop improvement, relatively few genetic and genomic resources are available for these species, and little is known on their genome organization, gene function, and regulatory networks. Recently, a Diversity Arrays Technology (DArT) array was constructed for potato [11]. The array contains markers derived from various *Solanum *species, including *S. bulbocastanum* and *S. commersonii*. DArT arrays offer the potential to simultaneously survey large numbers of anonymous loci distributed throughout the genome. DArT markers are highly transferrable across populations or even across species, since the DArT array comprises a structured marker set that is surveyed in each experiment. Importantly, polymorphic DArT markers correspond to a set of DNA clones that can be sequenced for downstream applications.

The availability of the potato DArT array together with the recent release of the complete genome sequences of cultivated potato [12] and tomato [13] provide an attractive opportunity for comparative genomic studies aimed at understanding genome evolution at the species level. The genomes of potato and tomato are largely syntenic, and molecular markers and gene content are predominantly conserved [13–16]. This degree of similarity has already enabled cross species comparative genomics approaches for gene mapping and cloning, reviewed by Bradeen [17]. Bioinformatics platforms improve community access to these resources and related *omics* collections, playing an important role for data mining and genome integration [18, 19]. In contrast to this wealth of knowledge and resources for cultivated potato and tomato, very little is known about genome structure and gene content in the wild relatives of potato.

In this paper, we exploited the reference genome sequences of potato and tomato and a collection of sequences of potato DArT array markers that show polymorphic representation across the genomes of *S. bulbocastanum* and/or *S. commersonii* genotypes. Our aim was to define a preliminary collection of marker sequences informative for the two species as a starting point for investigation of genome structure. This collection was also useful to highlight microscale genome sequence heterogeneity that possibly plays a meaningful role in functional and structural divergence between related species.

## 2. Materials and Methods

### 2.1. Plant Materials and DArT Marker Analyses

Two genotypes of *Solanum bulbocastanum *and two genotypes of *Solanum commersonii *were analyzed in this study. *S. bulbocastanum *genotypes include PT29 (PI243510), a source of the late blight resistance gene *RB *[3], and G15 (PI255516), a source of the *RB *locus allele *RB-rc *[20]. The *S. commersonii *genotypes include the frost tolerant cmm1T (PI243503) [8] and cmm6-3 (PI590886), a seedling genotype selected based on its crossability with cmm1T [21]. Total genomic DNA of individual plants for molecular marker analysis was isolated from fully expanded leaves from greenhouse-grown plants, following the protocol of Doyle and Doyle [22], with minor modifications. Two grams of leaf tissue were frozen in liquid nitrogen and ground in a mortar and pestle. Ground tissue was suspended in 6 mL lysis buffer (100 mM Tris-HCl pH 8.0, 20 mM EDTA, 2% CTAB, and 1.4 M NaCl) and incubated for 20 min at 65°C with occasional mixing by inversion. One volume of chloroform was added, and the tubes were mixed well and incubated at room temperature for 20 min with occasional inversion. Tubes were then centrifuged for 15 min at 1000 g, and the supernatant was transferred to a separate tube containing 2 volumes of 100% ethanol. Contents were gently mixed by inversion. Precipitated DNA was hooked out using sterile micropipette tips and transferred to 1.5 mL microfuge tubes. The DNA was washed twice with 75% ethanol and resuspended in TE (Tris pH 8.0 + 1 mM EDTA) buffer. DNA was shipped to Diversity Arrays Technology Pty Ltd. (Canberra, Australia) for DArT marker analysis.

Construction of the potato DArT array has been previously described [11]. The potato DArT array contains markers derived from *Solanum *species representative of the secondary and tertiary genepools of potato. Hybridization of genome representations from *S. bulbocastanum *and *S. commersonii *genotypes to the potato array and automatic calling of marker states were performed by Diversity Arrays Technology Pty Ltd. using established protocols [23]. Data that passed quality standards were analyzed for polymorphisms between genotypes within each species, and polymorphic markers were selected for downstream analyses. Clone cultures corresponding to each of these markers were robotically arrayed into a Whatman EasyClone 384 well plate (Whatman plc, Kent, UK) by Diversity Arrays Technology Pty Ltd. following manufacturer's instructions. Briefly, 10 *μ*L of each clone culture was applied to a well followed by air-drying of the plate. The FTA plates were then shipped to the University of Minnesota for PCR amplification and sequencing of clone inserts.

For clone insert PCR, 45 *μ*L of 10 mM Tris pH 8.0 + 0.1 mM EDTA was applied to each FTA plate well for 10 min at room temperature. PCRs were conducted in a 50 *μ*L volume that consisted of 1x PCR buffer (Applied Biosystems, Foster City, CA), 2.5 U of Amplitaq (Applied Biosystems), 200 *μ*M of each dNTP, 1 *μ*L of eluate from the FTA plates (as template), and 50 pmol of each primer (DArT-M13f: GTTTTCCCAGTCACGACGTTG and DArT-M13r: TGAGCGGATAACAATTTCACACAG; Integrated DNA Technologies (Coralville, IA)). Thermocycler (GeneAmp PCR System 2700 (Applied Biosystems)) conditions were 35 cycles of 94°C for 30 sec, 55°C for 30 sec, and 72°C for 30 sec followed by a single cycle of 75°C for 5 min. To each PCR, 5 *μ*L of 3 M NaOAC and 125 *μ*L of ice-cold ethanol were added. The PCR plates were stored at −20°C for at least one hour and then centrifuged at 2,500 g at 4°C for 30 min. The supernatant was gently poured off, and the open plates were centrifuged upside down at 800 g for 30 sec. To each tube, 175 *μ*L of room temperature 70% ethanol was added. The plates were again stored at −20°C and centrifuged as described above. Plates were dried completely at 37°C before adding 20 *μ*L of TE. Amplification was confirmed by agarose gel electrophoresis of 2 *μ*L of each purified PCR, staining with ethidium bromide, and visualization under UV light.

DNA sequencing of inserts was completed at the University of Minnesota BioMedical Genomics Center using BigDye Terminator (Applied Biosystems) cycle sequencing on an Applied Biosystems 3100 or 3700 automatic sequencer. Each sequencing reaction contained 1 *μ*L of purified PCR product and 3.2 pmol of DArT-M13f or DArT-M13r. Each insert was sequenced in both directions in separate reactions. Resulting sequences were trimmed of vector and assembled into consensus sequences using SeqMan, part of the DNASTAR (Madison, WI) Lasergene software package.

Out of 1,423 DArT marker clones sequenced, 756 hybridized in a polymorphic fashion with *S. bulbocastanum *genotypes and 550 hybridized in a polymorphic fashion with *S. commersonii *genotypes. Hereafter, these markers will be referred to as BLB- and CMM-specific markers, respectively. The remaining 117 DArT markers hybridized and were polymorphic in both species (indicated as BLB/CMM).

### 2.2. Sequence Analysis and Data Interpretation

The genome sequence of *Solanum phureja *[12] served as the reference genome for our analyses. The genome sequence of *Solanum lycopersicum* [13], another reference species among Solanaceae, was also employed. For both genomes, our analyses included 12 pseudomolecule sequences as well as unanchored scaffolds. We adopted gene annotations reported by the iTAG group (international Tomato Annotation Group) [24], assuring uniform annotation criteria and bioinformatics strategies and allowing coherent comparisons of the two reference genomes herein considered [13]. 

**Figure 1:**
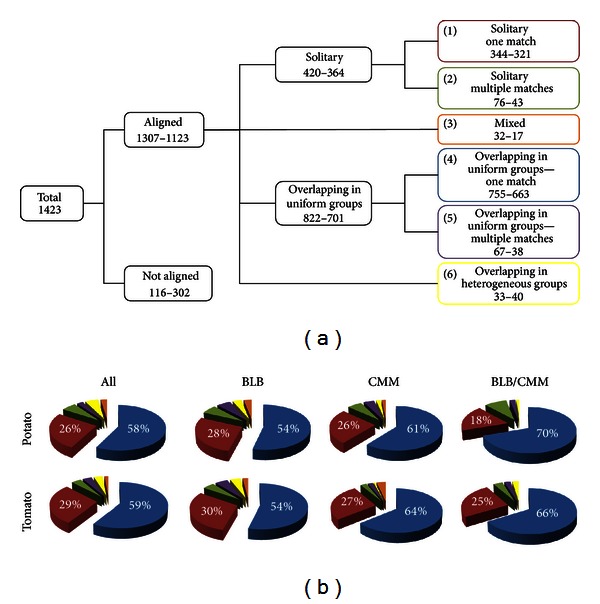
Categories of DArT markers alignments. (a) Values represent the number of alignments along the potato and tomato genome, respectively. (b) Pie charts of the percentage of aligned DArT markers, for each collection. The colour code is associated to the coloured rectangles of (a) and percentages are reported only when greater than 10%.

DArT marker sequences were aligned to the genome sequences using the splicing alignment software GenomeThreader [25] with 70% minimal nucleotide coverage and sequence identity. DArT alignments to genome sequences were grouped into six different categories (Figure 1(a)). A DArT marker sequence that aligned to a genome region independent of other DArT markers (i.e., one that does not overlap with any other marker sequences in the same genomic region) was classified as *solitary*. Each *solitary* marker was further subclassified as (1) *solitary one match*, if it aligned only once to the genome, or (2) *solitary multiple matches*, if it aligned more than once. A DArT marker whose alignment to the genome overlapped that of other DArT marker sequences was classified as an *overlapping *DArT. A DArT marker sequence having multiple matches to the genome, some of which are *solitary* and some of which are *overlapping*, was classified as subcategory (3) *mixed*. Other *overlapping* markers were further classified as *overlapping in uniform groups *when the group was composed of the same set of overlapping DArT marker sequences. This category comprised two subcategories: (4) *overlapping in uniform groups*—*one match* occurring only once in the genome and (5) *overlapping in uniform groups*—*multiple matches* appearing in two or more genome locations. DArT marker sequences which show multiple matches to the genome sequence and overlap sets of different DArT markers are defined as (6) *overlapping in heterogeneous groups*.

Fifty-three DArT marker sequences that did not align to either the potato or tomato genome sequences based on the GenomeThreader approach were assembled using CAP3 [26] (parameters: -p 40 –o 80) before a second alignment attempt based on BLASTn [27] (parameters: -e 0.003). These same DArT sequences were also aligned to the GenBank nucleotide collection (nr/nt) using BLASTn and to the nonredundant protein sequences dataset using BLASTp [28]. A BLAST2GO analysis [29, 30] was performed to classify genes associated to DArT marker sequences to show the cellular, biological, and molecular functional information of the subset annotation.

## 3. Results and Discussion

### 3.1. Dataset Description

The majority of the 1,423 DArT sequences analyzed have a length ranging between 350 and 850 nucleotides, providing a consistent dataset for subsequent bioinformatics analyses. In particular, 68% of BLB markers and 73% of CMM markers are 450 to 700 nucleotides in length (data not shown).

About 92% and 79% of all DArT sequences could be aligned the potato and tomato genomes, respectively (Table 1). These comprise 93% of BLB, 91% of CMM, and 90% of BLB/CMM DArT markers relative to the potato genome and 78% of BLB, 81% of CMM, and 76% of BLB/CMM DArT markers relative to the tomato genome. The discrepancy between the percentage of alignments to each genome is consistent with the composition of the reference potato DArT array that emphasizes markers from *Solanum *species more closely related to potato [11].

Sequence alignments were grouped into six categories, as described in Section 2. In the alignments to both potato and tomato genomes, DArT markers most frequently occurred as group (1) *solitary one match*, with 344 and 321 matches for potato and tomato, respectively, and as group (4) *overlapping in uniform groups-one match*, with 755 matches for potato and 663 matches for tomato (Figure 1(a)). For alignments to the potato genome, these two categories encompass 84% of all sequenced DArT markers: 82% for BLB, 87% for CMM, and 88% for BLB/CMM (Figure 1(b)). For alignments to the tomato genome, these same categories comprise 88% of all DArT marker sequences: 84% for BLB, 91% for CMM, and 91% for BLB/CMM. The remaining four marker alignment categories each represent less than 10% of the total number of aligned DArT marker sequences (Figure 1(b)). Briefly, groups (2) *solitary multiple-matches *and (5) *overlapping in uniform groups-multiple matches* show alignment to more than one genome region; this is probably due to repeated regions in the genome sequence; therefore, we considered these markers to be redundant. Groups (3) *mixed* and (6) *overlapping in heterogeneous groups* comprise DArT sequences with different alignment configurations probably due to intrinsic sequence properties. DArT marker sequences assigned to categories (1) and (4) localize in unique regions in both the potato and tomato genomes. Since these markers are associated unambiguously to specific genome locations, they were considered as nonredundant markers and were subjected to further analyses; DArT markers not assigned to alignment categories (1) and (4) were not considered further.

### 3.2. Analysis of Nonredundant DArT Markers

**Figure 2:**
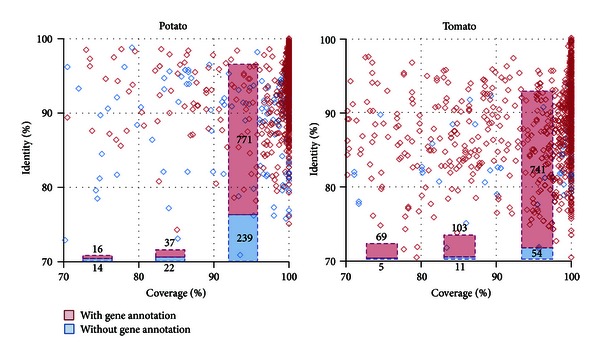
DArT marker sequences align predominantly with gene coding regions of the potato and tomato genome. The alignments associated (or not) to a gene locus along the potato and tomato genomes are highlighted in red (or blue). For each group, the number of alignments is also given.

**Figure 3:**
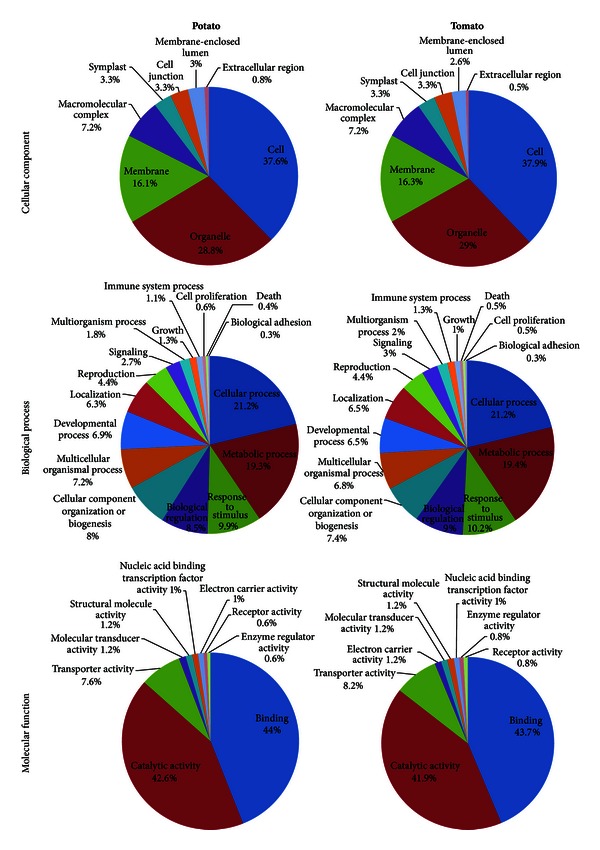
BLAST2GO analyses of the genes overlapping DArT marker regions.

In total 1,099 and 984 nonredundant (i.e., group (1) and group (4)) DArT marker sequences align to the potato and tomato genome sequences, respectively. The majority of the marker sequences aligns with a sequence identity exceeding 80% and a coverage greater than 90% (Figure 2). The percentage of alignments in the highest coverage category (between 90 and 100%) is 92% for potato and 75% for tomato. Many of the alignments overlap gene regions in both genomes (Figure 2). This is not unexpected since DArT markers are obtained through digestion by *Pst*I. *Pst*I is a methylation-sensitive enzyme; therefore, it is possible that it acts mainly on hypomethylated DNA which, in turn, may correspond to gene regions, which are typically hypomethylated [31]. In Figure 3, the BLAST2GO analyses of the genes overlapping DArT marker regions are shown for both potato and tomato annotations. In particular, the figure shows the overrepresentation of genes associated with catalytic and binding activities.

In percentage, the two marker groups (1 and 4) represent 84% and 88% of all markers sequences aligned to the potato and the tomato genomes, respectively. Interestingly, in contrast with average results across all DArT sequences (Table 1) showing more matching DArT sequences to potato than to tomato, a higher proportion of the nonredundant groups align to the tomato genome than to the potato genome. This may be due to the higher contribution of ambiguous alignments (group (2) and (5)) in potato. This in turn suggests a higher sequence repetitiveness in the potato genome or better sequence quality for the tomato genome [12, 13]. Overall, nonredundant DArT marker sequences show very high coverage in potato compared to tomato (Figure 2), confirming higher phylogenetic similarity amongst potato species.

We next examined total coverage of the genome sequences from cultivated potato and tomato represented by alignments with DArT marker sequences (Table 2). Details per chromosomes are reported in the supplementary Table S1 (see Table S1 in Supplementary Material available at http://dx.doi.org/10.1155/2013/257218). In general, BLB DArT markers encompass a greater number of nucleotides in each genome than CMM or BLB/CMM markers. This is not surprising since BLB markers are the largest subset of DArT markers examined in this study. BLB DArT markers represent 208.8 Kbp of the potato genome but only 175.8 Kbp of the tomato genome. In contrast, CMM and BLB/CMM markers represent approximately equivalent regions of the potato and tomato genomes (CMM: 137.9 Kbp for potato versus 139.6 Kbp for tomato; BLB/CMM: 29.4 Kbp for potato versus 24.7 Kbp for tomato). We further divided the nonredundant DArT markers into two subclasses. *Common *markers align with the genome sequences of both potato and tomato; *specific *markers align to only one of the two genomes (Table 2). Within each sub-class, alignments were either *ungapped* (i.e., marker sequences aligned to genome sequences without disruption) or *gapped* (i.e., marker sequences aligned to genome sequences but alignments were interrupted by genome sequence not found in marker sequences). It is noteworthy that the same DArT marker sequence could be *ungapped* when aligned to the potato genome and *gapped* when aligned to the tomato genome or *vice versa*. The relative ratio of *gapped* versus *ungapped* regions of all BLB, CMM, and CMM-BLB DArT marker sequences relative to the potato and tomato genome sequences provides insight into patterns of genome evolution and species relationships. Distinction between *gapped* and *ungapped* alignments is necessary since variability in the length of *gapped *markers can complicate interpretation of the degree of genome coverage by the marker sequences. In potato, for example, the size of most of the gaps (89%) ranges from 20 to ~1000 bps. The remaining ones reach a maximum at ~5000 bps (not shown). For *common* DArT markers, the contribution of *ungapped* regions to total genome representation is higher in potato than in tomato for each marker collection. In contrast, for *common *markers, the contribution of *gapped* regions is generally lower in potato than in tomato. This again reflects higher phylogenetic similarity of the wild species to the cultivated potato. However, it is interesting to note that the relative frequency of *common gapped* regions compared to *common ungapped* ones in potato versus tomato is comparable for both BLB (14.21% in potato and 16.68% in tomato) and BLB/CMM (5.14% potato and 3.16% in tomato) DArT markers. The frequency of CMM *common gapped* and *ungapped* regions differs in potato (7.73%) with respect to tomato (15.64%). This indicates that, in contrast to BLB markers, CMM markers align with fewer gaps to the potato genome sequence than to the tomato genome sequence. This implies that the genomes of *S. commersonii *and potato are more similar at a DNA sequence level than are the genomes of *S. bulbocastanum* and potato, consistent with *S. commersonii *being phylogenetically more closely related to potato than is *S. bulbocastanum*, as the analyses based on plastid genomes previously suggested [32–34].

Considering the contribution of *specific* DArT markers, *ungapped *BLB markers provided the greatest overall genome coverage for both potato and tomato, consistent with higher representation of BLB markers in our dataset (Table 2). Importantly, the relative proportion of *gapped* regions compared to *ungapped* regions for the *specific* alignments indicates a comparable behaviour in the three marker collections in both species.

### 3.3. Genome Sequence Heterogeneity

We compared marker origins and alignment classifications across the potato and tomato genomes (Table 3). In general, the majority of aligned DArT markers are *ungapped* in both potato and tomato: 328 (77%) for BLB, 297 (83%) for CMM, and 65 (91%) for BLB/CMM. Eight BLB and 16 CMM markers align to both genomes in a *gapped* configuration (Table 3). Interestingly, a high percentage of aligned markers exhibit heterogeneous behaviours across the potato and tomato genomes (i.e., *gapped* versus *ungapped *in potato versus tomato and vice versa). These sequences are a source of marker variability between wild and cultivated species that can be exploited in future studies.

Seven BLB, 16 CMM, and one BLB/CMM markers aligned to the genomes of both potato and tomato in a *gapped *configuration (Table 3). As shown in Table S2, each of the seven BLB DArT markers aligned to gene regions in both species. Among these, five regions corresponded to genes with identical annotations in potato and tomato. On the other hand, among the 16 CMM DArT markers, only 10 and 14 aligned to gene coding regions in potato and tomato, respectively. Of the 10 CMM markers aligning to both potato and tomato gene coding regions, all of the 10 aligned to regions with identical gene annotations in both species (Table S2).

Nine DArT markers, three from BLB and six from CMM, aligned with the same alignment structure (i.e., number and length of *gapped* and *ungapped* regions) to homologous chromosomes in both potato and tomato and to gene loci with the same annotation (Table S2). The remaining two BLB, four CMM, and one BLB/CMM markers, although aligning to homologous chromosomes in genes of the same annotation, showed heterogeneous (i.e., number and length of *gapped* and *ungapped *regions) alignment structure (Table S2). These observations of microscale genome heterogeneity may be relevant to investigation of genome structures, functionalities, and properties of the represented *Solanum* species.

### 3.4. DArT Marker Sequences Not Aligned to the Reference Genomes

Some DArT markers could not be aligned to one or both genome sequences (Table 1). In particular, 116 marker sequences could not be aligned to the potato genome, 302 marker sequences could not be aligned to the tomato genome, and 51 marker sequences could be aligned to neither to potato nor to tomato. These were selected as putative wild species-specific markers and were assembled using the CAP3 software, yielding seven assembled consensus sequences comprising 20 sequences in total. The remaining 31 DArT marker sequences could not be assembled. Next, we attempted a less stringent alignment of the resulting 38 sequences (31 unassembled sequences plus seven consensus sequences) to the potato and the tomato genome sequences using the BLASTn algorithm (Table S3). Using this approach, 18 DArT marker sequences could be assigned to single locations in both the potato and tomato genomes, and only nine markers aligned to multiple genome locations in one or both species. In these cases, the less stringent alignment search performed by the BLAST software helped to confirm the presence in the potato and tomato genomes of 27 DArT marker sequences, previously unidentified in the more stringent GenomeThreader analysis. Moreover, in some cases, the BLASTn analysis confirmed matches to the same chromosome for both potato and tomato (e.g., DArT markers 472847 (chromosome 1), 537586 (chromosome 8), 473780 (chromosome 2), and 534573 (chromosome 11)). The presence of low level sequence similarity between these markers and the potato or tomato genome sequences revealed distant relationships between the wild and cultivated species and may be exploited in the study of cross-species genome heterogeneity. Twenty-two DArT markers (Table S3) showed extreme repetitive distribution along the potato and tomato chromosomes and were described by ambiguous annotations. Nevertheless, protein-based annotations (BLASTp), when present, generally confirmed homology with *Solanum* proteins or with those from more distantly related plant species. Two DArT marker sequences failed to align to the genomes of either potato or tomato even under more permissive analytical criteria.

## 4. Conclusions

Potato (*S. tuberosum*) and tomato (*S. lycopersicum*) belong to the subgenus *Potatoe *of the large and diverse genus *Solanum*. Although horticulturally distinct, potato and tomato share a clear evolutionary history that is well supported by molecular data [35, 36]. The species are thought to have diverged from a common ancestor approximately 6.2 to 7.3 million years ago [37, 38]. Sexual isolation and subsequent divergence of the two species were accompanied by a series of structural genomic changes including chromosome arm inversions and large-scale translocations [14, 15]. Nevertheless, the genomes of potato and tomato are largely syntenic and molecular marker and gene content are predominantly conserved [14–16]. This degree of similarity has enabled cross species comparative genomics approaches for gene mapping and cloning, reviewed by Bradeen [17], efforts that will likely be furthered by the recent release of the complete genome sequences of potato [12] and tomato [13].

In this study, we proposed a suitable methodology to exploit partial genome information from wild species in the presence of reference genomes from related species. This approach, here exploited with DArT marker sequences, can also be employed in partial genome resequencing or similar efforts. Our results also highlighted the presence of divergent sequence relationships and heterogeneous alignment structures, including the presence/absence of gaps, which are detectable thanks to appropriate, less stringent comparative methods. This divergence commonly occurred even in gene pairs with apparent orthologous relationships and presumed functional conservation, and it could often be confirmed both in potato and tomato genomes. Evidence from results supported by two reference-related species partially overcomes possible limits that may be due to the quality of first released genomes and suggests a fine microscale genome structural divergence between wild and cultivated species in the Solanaceae. Our results confirm the utility of suitable analytical approaches that could be applied when partial genome information is available, capable of highlighting genome microscale variability that, although often occurring at the gene level, is not detectable when investigating genome functionality at transcriptome and proteomic levels. 

## Supplementary Material

Table S1 – Classification of Common DArT markers aligned with gaps. Circles in the first column represent DArT marker sequences aligning to regions associated with loci with the same annotation in both genomes, with the same [white] or different [black] gene structures; unannotated DArT marker sequences and those with incoherent annotation are represented as grey triangles.
Table S2 – Additional BLAST annotation for unmapped DArT marker sequences. When DArT marker sequences are assembled into contigs, the contig ID is reported in the second column.
Table S3 – Number of nucleotides (in Kbp units) covered by DArT alignments per chromosome. For details on coverage categories see Materials and Methods.

## Figures and Tables

**Table 1:** Results of DArT alignments to potato and tomato reference genomes. For each collection, the total number of DArT markers and the number (%) of aligned DArT markers are reported.

Collection	Total no. of DArT	No. aligned (%) to	
		Potato	Tomato
BLB	756	703 (92.9)	586 (77.5)
CMM	550	499 (90.7)	446 (81.1)
BLB/CMM	117	105 (89.7)	89 (76.1)

All	1423	1307 (91.8)	1121 (79.0)

**Table 2:** Number of nucleotides (in Kbp units) covered by DArT alignments. For details on coverage categories, see Section 2.

Coverage category		Potato			Tomato	
	BLB	CMM	BLB/CMM	BLB	CMM	BLB/CMM
Common						
Ungapped	132.3	97.7	20.5	128.2	95.3	19.1
Gapped	18.8	7.6	1.1	21.4	14.9	0.6
Specific						
Ungapped	46.1	22.3	7.7	22.5	17.3	4.4
Gapped	10.2	10.4	0.2	3.7	12.1	0.7

Total	208.8	137.9	29.4	175.8	139.6	24.7

**Table 3:** Comparison between DArT alignments to potato and tomato genomes. Number of DArT markers aligned along the potato (horizontal) and tomato (vertical) genomes for each collection, given in parenthesis. Each cell, within each matrix, shows the number of DArT markers per alignment type: ungapped, gapped, or not aligned.